# Recent Advances in N-O Bond Cleavage of Oximes and Hydroxylamines to Construct N-Heterocycle

**DOI:** 10.3390/molecules28041775

**Published:** 2023-02-13

**Authors:** Hui-Min Jiang, Yi-Lin Zhao, Qing Sun, Xuan-Hui Ouyang, Jin-Heng Li

**Affiliations:** 1 Key Laboratory of Jiangxi Province for Persistent Pollutants Control and Resources Recycle, Nanchang Hangkong University, Nanchang 330063, China; 2 State Key Laboratory of Applied Organic Chemistry, Lanzhou University, Lanzhou 730000, China; 3 State Key Laboratory Base of Eco-Chemical Engineering, College of Chemical Engineering, Qingdao University of Science and Technology, Qingdao 266042, China; 4 School of Chemistry and Chemical Engineering, Henan Normal University, Xinxiang 453007, China

**Keywords:** N-O bond cleavage, oximes, hydroxylamines, N-hetercycles, transition-metal catalysis, radical reactions

## Abstract

Oximes and hydroxylamines are a very important class of skeletons that not only widely exist in natural products and drug molecules, but also a class of synthon, which have been widely used in industrial production. Due to weak N-O σ bonds of oximes and hydroxylamines, they can be easily transformed into other functional groups by N-O bond cleavage. Therefore, the synthesis of N-heterocycle by using oximes and hydroxylamines as nitrogen sources has attracted wide attention. Recent advances for the synthesis of N-heterocycle through transition-metal-catalyzed and radical-mediated cyclization classified by the type of nitrogen sources and rings are summarized. In this paper, the recent advances in the N-O bond cleavage of oximes and hydroxylamines are reviewed. We hope that this review provides a new perspective on this field, and also provides a reference to develop environmentally friendly and sustainable methods.

## 1. Introduction

N-O bond units, such as oxime ethers, and oxime esters and hydroxylamines, are not only common components of natural products and drug molecules, but also play an important role in organic synthesis and the construction of heterocycles, which have received wide attention [1,2,3,4]. Due to weak N-O σ bonds [5], the average energy of about 57 kcal/mol^−1^, is far below the σ C-X (X = C, N, O), thus N-O bond units have been considered to be quite unstable bonds. Those N-O bond compounds are easy to break under the influence of a transition-metal catalyst, transition-metal-free catalyst, or photocatalyst, allowing the formation of new C-N, C-O, or C-C bonds to build nitrogen compounds (e.g., nitrogen-containing heterocycles [5,6,7,8,9], alkyl nitriles [10,11,12], and amines [13]), or oxygen-containing compounds [14,15,16,17,18]. Reactions involving N-O bond cleavage have a long history in organic synthesis. For example, the classic Beckmann rearrangement reaction has been widely used in organic synthesis and industrial, especially for the synthesis of Nylon [19]. Recently, the transition metal-catalyzed C-H functionalization/cyclization reactions have also been well-established for efficient access to the N-heterocycles, such as aziridines, β-lactam, 1*H*-indazoles, oxazoles, pyrrolines, pyrroles, pyridines, and other cyclic compounds [5,6,7,8,9]. It is worth noting that, in 2014, Jiang’s group reviewed the transition metal-catalyzed C-H functionalization of oximes’ internal oxidant [8]. However, the development of selective N-O bond cleavage strategies and organic synthesis strategies involving radical-intermediates remains one of the hotspots and difficulties.

Oximes, including hydroxy oximes, oxime ethers, and oxime esters, are easy to prepare and transform units as nitrogen-containing and oxygen-containing versatile building blocks in organic synthesis, and because of their safety, cheap and other advantages, are widely used in the laboratory and industrial applications [20,21,22]. Oximes have reactive N-O bonds, and the repulsion between the lone-pair electrons of its adjacent N and O atoms leads to the easy fracture of N-O bond. Moreover, it has a strong oxidation capacity, and can construct nitrogen heterocyclic compounds without the addition of an oxidant. In addition, combining the N-O bond cleavage and C-H bond activation could construct complex compounds by using the oxidation property of the N-O bond, and C-C bond [23]. 

Hydroxylamine is a valuable component of many agricultural chemicals, pharmaceuticals, and natural products. Hydroxylamine is a stable, easily prepared, and versatile amination reagent, which could not only act as a nucleophile, but also converts the hydroxyl group into a good leaving group and acts as an electrophile. Changing the substituents on the two electronegative atoms of hydroxylamine allows more diverse modifications and the introduction of functional groups [24,25,26,27,28]. It can also modulate the ability of both the electrophilic and nucleophilic properties, thereby regulating the activity of the reaction. 

Recently, radical-mediated cyclization and functionalization of oximes or hydroxylamines have been achieved to construct the C-C bonds, C-N bonds, C-O bonds, and *N*- heterocycles [15,16]. Notably, Wu [9], Zeng [10], and others [20] have recently summarized cycloketone oximes as novel and practical precursors for the synthesis of alkyl nitriles, however, these reviews are limited to photochemical radical reactions. Therefore, this paper focuses on the latest progress in N-O bond cleavage strategies to construct N-containing heterocyclic compounds by using hydroxy oximes, oximes esters, hydroxylamines, and related compounds.

## 2. The N-O Bond Cleavage of Oximes to Construct N-Heterocycle

### 2.1. Hydroxy Oximes

**Figure 1:**
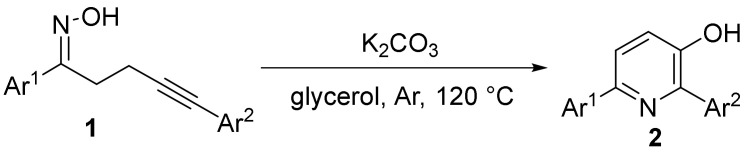
The N-O bond cleavage of γ-δ-alkynyl oximes to pyridinol.

In 2017, the Guan research group [29] developed a K_2_CO_3_-mediated γ-δ-alkynyl oximes **1** cyclization rearrangement reaction to synthesize pyridinol **2** (Figure 1). The internal nucleophilic addition of oximes to acetylene is cyclized under mild conditions, followed by efficient rearrangement of in situ o-vinyloxime intermediates. This reaction uses readily available starting materials, tolerates a wide range of functional groups, and synthesizes a variety of pyridines derivatives with good yields that are challenging to synthesize.

**Figure 2:**
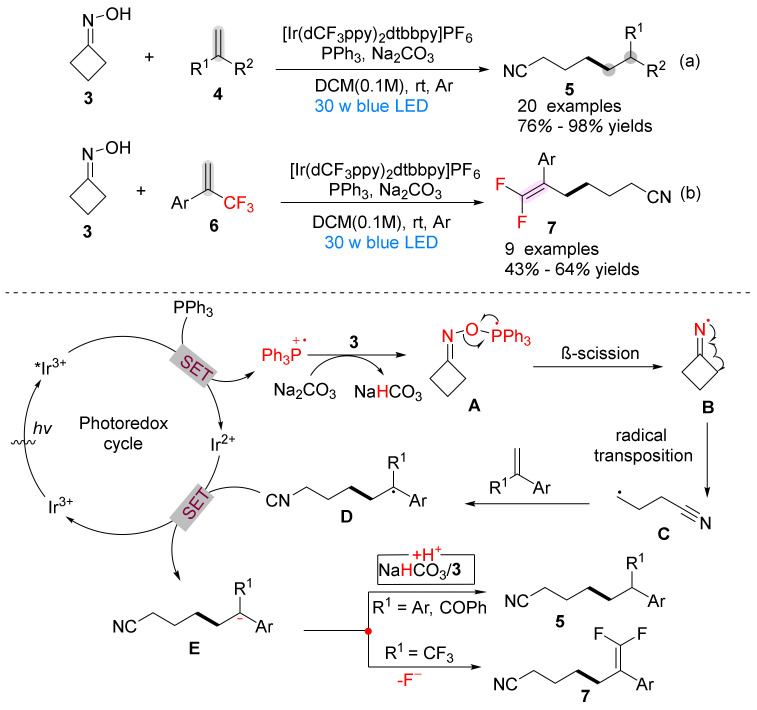
Ir-catalyzed N-O bond cleavage to form cyano compounds and proposed mechanism.

In 2019, Yang’s [30] group reported the generation of a series of functional cyano compounds **5** (Figure 2a) and cyanodifluorostyrene **7** (Figure 2b) under the action of a photocatalyst, PPh_3_ and a base, using cyclobutanone oxime **3** as a raw material and alkene **4** and α-trifluoromethyl alkene **6** with different electronic and structural characteristics as effective radical acceptors. This is a new method to photo-induce phosphoacyl radical directly mediated cycloketoxime N−O cleavage developed through a polarity/SET crossover process.

The author further proposed a possible reaction mechanism for the synthesis of cyano compounds and cyanodifluorostyrenes by Ir-catalyzed cyclobutyrooxime ketones with olefins and α-trifluoromethyl olefins (Figure 2): *Ir(III) was obtained under light irradiation, PPh_3_ was oxidized to triphenylphosphine cation, and **3** was combined with it under the action of Na_2_CO_3_ to obtain **A**. **A** underwent β-splitting to achieve N-O bond cleavage and deoxygenation to obtain **B**, **B** underwent C-C cleavage to obtain cyanoalkyl radical **C**, and combined with olefins to obtain **D**. Then, **D** was reduced by Ir(II) to obtain **E**. The intermediate radical **E** was protonated to obtain cyano compounds **5**, and **E** was combined with CF_3_-styrene to undergo *β*-F elimination to obtain product **7**.

**Figure 3:**
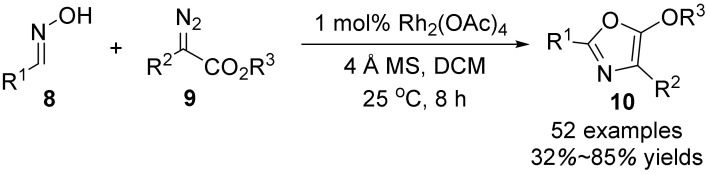
Rh-catalyzed N-O bond cleavage to form oxazoles.

In 2021, Wang’s group [31] reported the [3+2] cyclization reaction of rhodium-catalyzed oximes **8** and α-diazo-carbonyl compounds **9** (Figure 3). Under controlled conditions with Rh_2_(OAc)_4_ as catalyst, the addition of a dehydrating agent and using DCM as solvent, the oxazoles derivatives **10-1** were generated with good yield at room temperature for 8 h. In addition, the generation of oxime ethers was achieved in the absence of Rh catalyst under blue LED.

**Figure 4:**
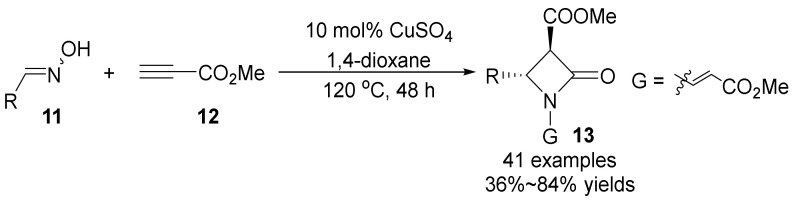
Cu-catalyzed N-O bond cleavage to form lactams.

In the same year, Wang’s group [32] reported that copper(II)-catalyzed oximes **11** and methyl propionates **12** construct *trans*-configuration β-lactam **13** and cyclocycloβ-lactam (Figure 4). This scheme has excellent substrate flexibility and diastereoselectivity (up to >99:1 dr). Based on the initial 1,3-Aza proton transfer of oximes and methyl propionates, a copper (II)-catalyzed scheme for the construction of multisubstituted β-lactam was developed and yielded the transformable *β*-lactam with high diastereoselectivity.

### 2.2. The N-O Bond Cleavage of the Oxime Esters to Construct 2H-Azirines

**Figure 5:**
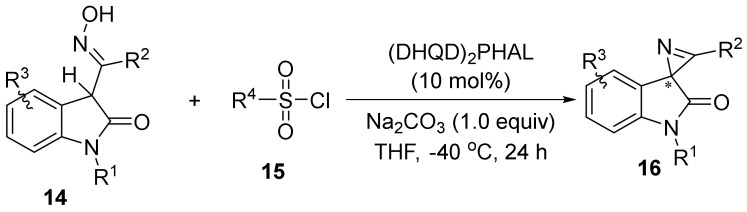
Organocatalytic asymmetric N-O bond cleavage to form spirooxindole 2*H*-azirines.

In 2016, Yuan and Xu’s group [33] demonstrated the example of enantioselective synthesis of chiral spirooxindole 2*H*-azirines **16** through the Neber reaction (Figure 5). The cyclization reaction of 3-O-sulfonyl ketoxime, in situ generated from isatin ketoxime **14** and sulfonyl chloride **15**, delivers the spirooxindole 2*H*-azirines by using (DHQD)2PHAL as the catalyst. With the established protocol, a range of chiral spirooxindole 2*H*-azirines could be obtained in good to excellent yields with up to a 92:8 enantiomeric ratio.

**Figure 6:**
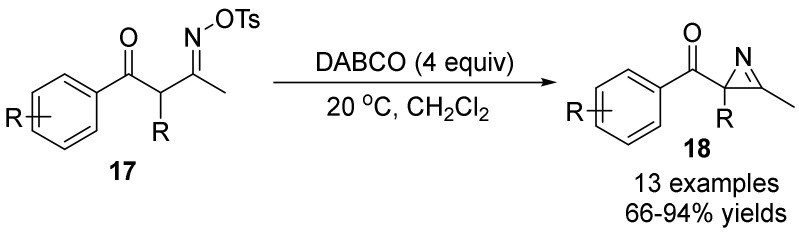
Base-promoted N-O bond cleavage to form 2*H*-azirines.

In 2017, Ohwada’s group [34] reported that 3-sulfonyloxyimino-2-methyl-1-phenyl-1-butanones **17** could provide different intramolecular cyclization products under different conditions (Figure 6). The reaction of oximes under base (DABCO, 1,4-diazabicyclo[2.2.2]octane) indicated that intermediacy of neutral enol to afford 2H-azirine **18**. It is noteworthy that the target product **18** is successfully converted into isoxazoles and oxazoles under heating conditions. This method suggests generally preferred trajectories for the 5-trig and 3-trig cyclization reactions with an oxime functionality.

**Figure 7:**
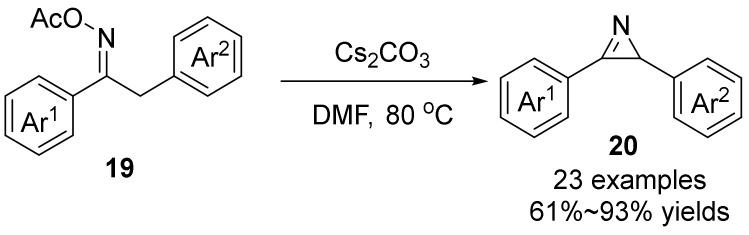
Base-promoted N-O bond cleavage to form 2*H*-azirines.

In 2018, Guan’s group [35] demonstrated a modular 2*H*-azirines **20** synthesis from ketoxime acetates **19** via Cs_2_CO_3_-mediated cyclization (Figure 7). Intramolecular cyclization of Ketoximes and their derivatives to 2H-azirines is a simple and practical process due to their being readily accessible, stable to air and moisture, and nontoxic characteristics. Compared to traditional methods, no explosive azides and no side effects make the method more efficient and safer. In addition, this method has scale-up applicability because it can perform a gram-scale reaction, and the synthesized 2*H*-azirines can be efficiently converted to various azaheterocycles.

**Figure 8:**
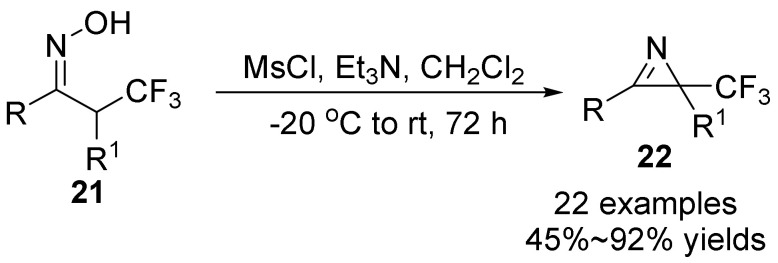
Base-promoted N-O bond cleavage to form trifluoromethyl-containing 2*H*-azirines.

In 2018, Ma’s group [36] developed a one-pot metal-free Neber reaction to synthesize trifluoromethyl-azirines **22** by using the aryl/alkyl-2,2,2-trifluoroethylketoximes **21** (Figure 8). This is the first time trifluoromethyl-containing 2*H*-azirines have been constructed by the Neber reaction, and form highly functionalized azirines with trifluoromethyl group by only using Et_3_N and MsCl under mild conditions. In addition, further transformations of azirines to CF_3_-containing pyrrole and amino-alcohol derivatives are also demonstrated.

### 2.3. The N-O Bond Cleavage of the Oxime Esters to Construct Five-Membered N-Heterocycle

**Figure 9:**
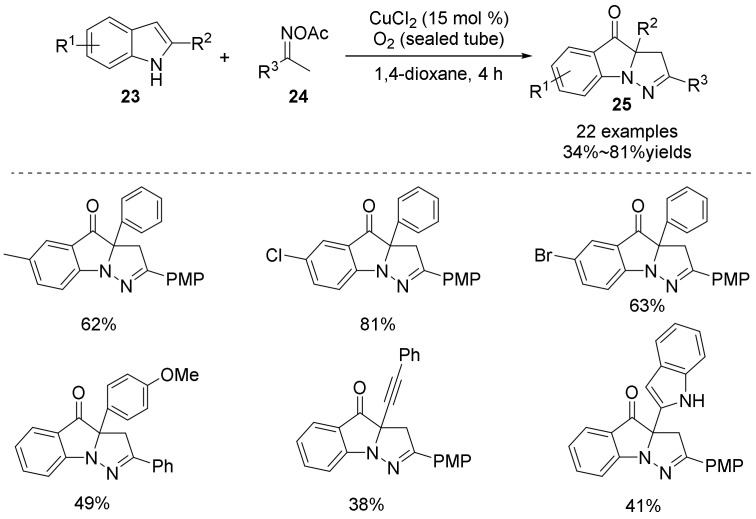
Cu-catalyzed N-O bond cleavage to indoles derivatives.

In 2015, Huang and Deng’s group demonstrated copper-catalyzed internal oxidant-triggered cyclization of indoles **23** with oxime acetates **24** under oxygen (Figure 9) [37]. The method is an elegant way to synthesize N-1, C-2, and C-3 trifunctionalization of indoles **25** in one-pot with wide substrate scope and good functional group tolerance. A range of indoles was compatible with this system. In particular, the functional groups of alkyne groups, halogens and alcohols on the indole skeleton are of great significance and can be further transformed and modified to synthesize more complex indole skeleton molecules. Mechanistic studies have shown that this reaction proceeds through a radical process, and the use of oxygen makes the reaction environmentally friendly and easy to operate.

**Figure 10:**
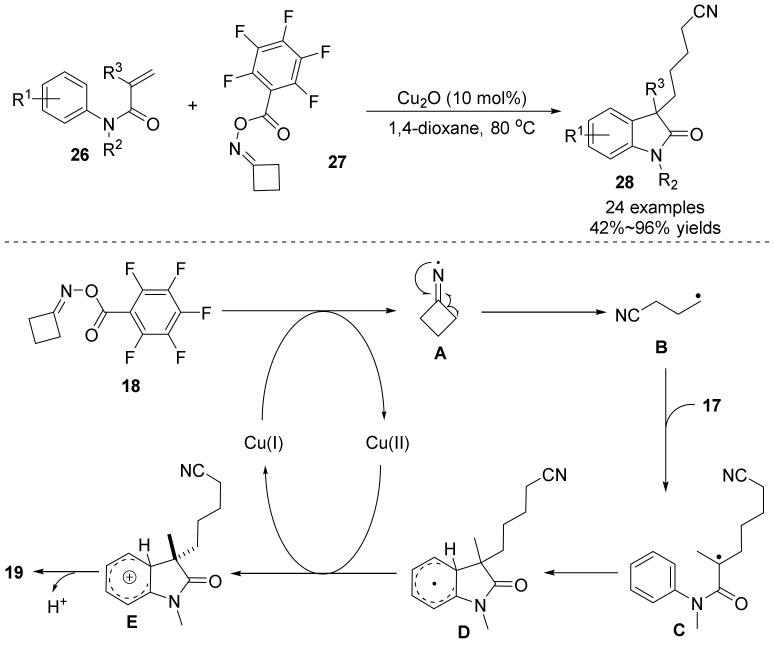
Copper-catalyzed cyanoalkylarylation of alkenes to form oxindoles and dihydroquinolin-2(1*H*).

In 2018, Guo’s group demonstrated copper-catalyzed cyclization of acrylamides **26** with cyclobutanone *O*-acyl oximes **27** under redox-neutral conditions (Figure 10), wherein C(sp^3^)-C(sp^3^) and C(sp^3^)-C(sp^2^) bonds were simultaneously formed in a one-step reaction [38,39]. The method has a wide range of substrates and good functional group tolerance and is an effective way to synthesize a variety of cyanoalkylated oxindoles. Mechanistic studies show that the radical inhibitors (TEMPO) captured the alkyl radical **B**, indicating that this reaction may be conducted via a radical process. Therefore, the authors propose the mechanism of the reaction, first the interaction of Cu(I) and cyclobutanone *O*-acyl oximes to form the Cu(II) and alkyl radical **A,** followed C–C bond cleavage to generate the radical **B**. Then, radical **B** was added to the alkenes, a sequential cyclization and SET processes were involved to afford the target products **28**, and the Cu(I) regenerated to complete the catalytic cycle.

**Figure 11:**
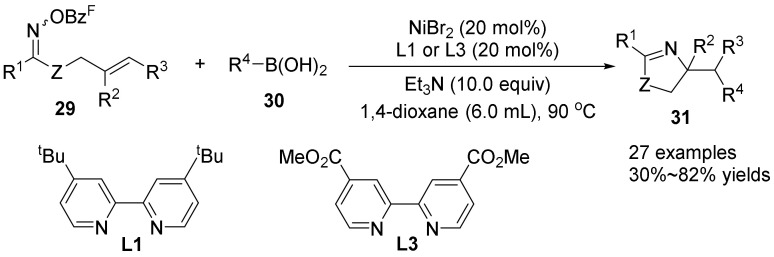
Ni-catalyzed N-O bond cleavage to form pyrroline.

In 2017, Selander’s group [40] reported a nickel-catalyzed 1,2-aminoarylation reaction of oxime ester hydrocarbons **29** with alkyl boronic acids **30**. The author used 20 mol% NiBr_2_ as a catalyst, 1.4-dioxane as a solvent, and reacted at 90 °C to obtain a variety of pyrroline derivatives **31** in moderate to good yields (Figure 11). Through the continuous formation of C(sp^3^)-N bond and C(sp^3^)-C(sp^2^) bond, a variety of pyrroline derivatives were synthesized in good yields. The use of nickel catalysis can overcome the long-term problem of β-H elimination in cross-coupling reactions, and change the selectivity by changing the substrate, ligand or metal, and finally provide pyrroline as the main product. The reaction has a wide range of substrates, and different alkyl boronic acids substrates are compatible. However, when cycloketone oxime esters are used as substrates, pyrroline compounds are not obtained, but nitrile compounds are generated, and the scheme is also extended to imidazoline compounds.

**Figure 12:**
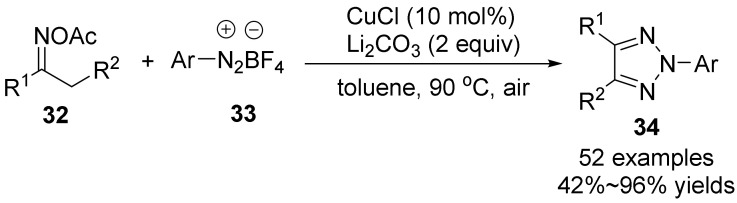
Cu(I)-catalyzed N-O bond cleavage to form 1,2,3-triazoles.

In 2017, Jiang’s group [41] studied the copper-catalyzed coupling of oxime acetate **32** with tetrafluoroborate aryldiazonium salts **33** to synthesize N-2-aryl-1,2,3-triazoles **34** in moderate to good yields (Figure 12). Under copper catalysis, the C-N2 bond cleavage of aryl diazonium salts readily reacts with aryl radicals to form the classic Meerwein arylation reaction. In this reaction, the use of p-methoxyphenyldiazotetrafluoroborate as a complex favors the single electron transfer process of the N-O bond cleavage of oxime acetate and inhibits the Meerwein arylation process. This strategy does not require the use of potentially explosive azides, consequently reducing the risk. The mild reaction conditions, high yields, wide range of substrates, weight scalability, and high yield of target products make it suitable for the modification of various natural products.

**Figure 13:**
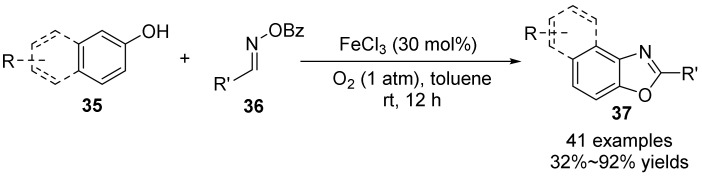
Fe(III)-catalyzed N-O bond cleavage to form benzoxazole.

In 2017, Zeng’s group [42] disclosed an new method for the synthesis of polysubstituted benzoxazole derivatives **37** in moderate to extra-high yields with aid of iron (III) salts at room temperature by using simple and readily available phenol derivatives **35** as precursors to react with benzaldoxime **36** (Figure 13). The benzoylaldoxime acts not only as a substrate, but also as an auxiliary ligand supporting the iron salt to promote the transformation, so that the reaction proceeds more easily. The mild and simple reaction conditions, low cost, and high efficiency broadens the method of synthesizing benzoxazole derivatives with various structures.

**Figure 14:**
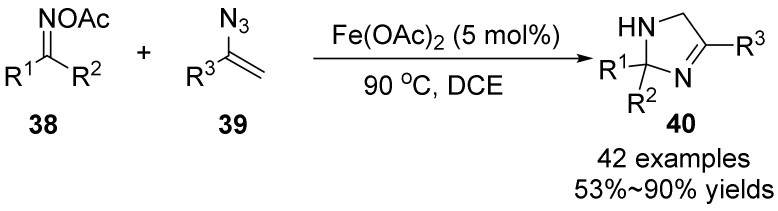
Ir-catalyzed N-O bond cleavage to form 2*H*-imidazole.

In 2017, Deng’s group [43] studied the synthesis of 2*H*-imidazole **40** by [3+2] cyclization of oxime acetate **38** and ethylene azide **39** under redox-neutral conditions (Figure 14). The scheme is carried out under mild reaction conditions without additives or ligands. Iron is used as a catalyst and oxime acetate is used as an internal oxidant to break the N−O/N−N bond to form two new C−N bonds, which provides a fast way for a series of functionalized 2*H*-imidazoles. This method has the advantages of low catalyst cost, simple conditions, convenient operation, high atom economy, mild condition and good functional group tolerance. Various 2,2,5-trisubstituted 2*H*-imidazoles were synthesized in good yields. The synthesis of functionalized 2*H*-imidazole is rarely reported, which provides a very desirable method for the synthesis of functionalized 2*H*-imidazole.

**Figure 15:**
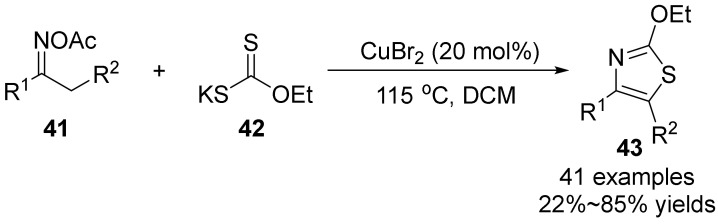
Cu(II)-catalyzed N-O bond cleavage to form thiazol-2-yl ether.

In 2018, Jiang’s group [44] established a copper-catalyzed [3+2] cyclization reaction of oxime acetate **41** and xanthate **42** to synthesize thiazol-2-yl ether **43** (Figure 15). This method also uses oxime acetate as the internal oxidant, involving the oxidative cleavage of N-O bond and the activation of vinyl C-H bond. The target product is synthesized with good stereoselectivity and functional group tolerance, which has certain practicability.

**Figure 16:**
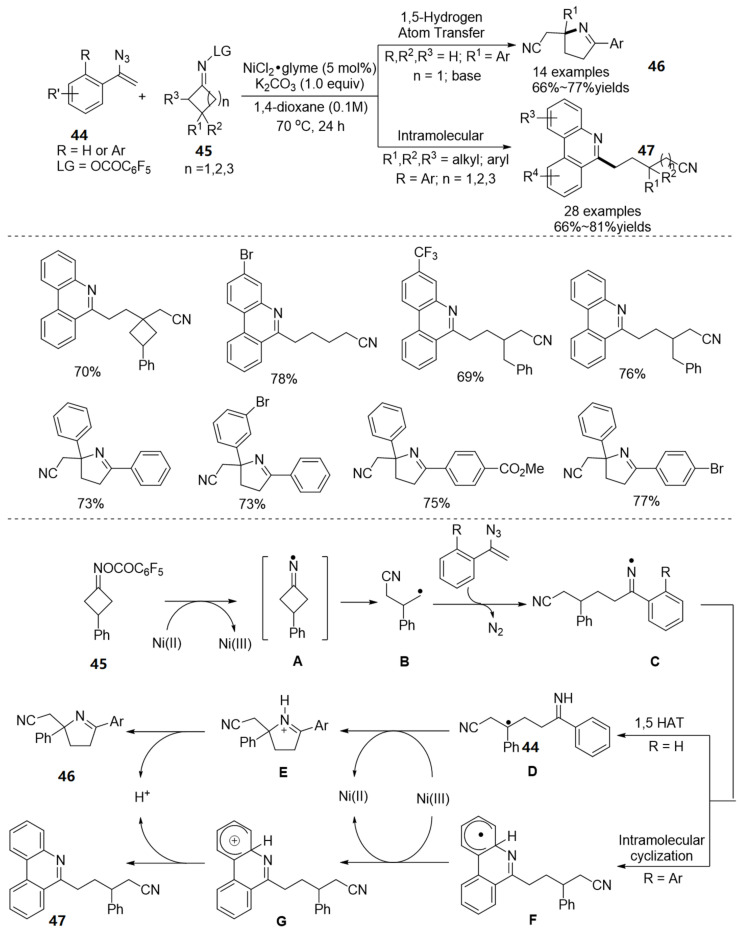
Ni-catalyzed N-O bond cleavage to form cyanoalkylated 3,4-dihydro-2*H*-pyrroles and phenanthridines.

In 2019, A Ni-catalyzed diversity of new methods for the synthesis of cyanoalkylated 3,4-dihydro-2*H*-pyrroles **46** and phenanthridines **47** through ring-opening/radical addition/ring-closing cascade of cycloketone oxime esters **45** and vinyl azides **44** have been developed by Guo’s group (Figure 16) [45]. The reaction proceeds to produce the multifarious N-heterocycles in good yields via adjustment of the substrate’s properties in the presence of Ni(II) catalyst and K_2_CO_3_ at 70 °C, thereby featuring environmentally friendly properties under mild and redox-neutral conditions. This strategy generated two types of iminyl radicals that are characteristic of the reaction.

Based on the control experiments, the author proposed a plausible mechanism (Figure 12, bottom). First, the cycloketone oxime ester is reduced by Ni(II) catalyst to give Ni(III) and the iminyl radical **A**, followed undergoes C-C bonds fragmentation to produce γ-cyanoalkyl radical **B**. Subsequently, the radical intermediate **B** attacks the azides **35** to generate a new iminyl radical **C**. On the one hand, when the R substituent is hydrogen, iminyl radical **C** undergoes a 1,5-hydride shift to give a new alkyl radical **D**. An intramolecular cyclization occurs to afford intermediate **E** with the aid of Ni(III) through single electron transfer (SET), and the regeneration of Ni(II) complete the catalytic cycle. Intermediate **E** is transformed into the target product **37** via deprotonation. On the other hand, when the R substituent is aryl, intramolecular cyclization of the intermediate **C** occurs to deliver the intermediate **F**, followed oxidize to intermediate **G** by Ni(III) through single electron transfer (SET), and the Ni(II) is also generated to complete the catalytic cycle.

**Figure 17:**
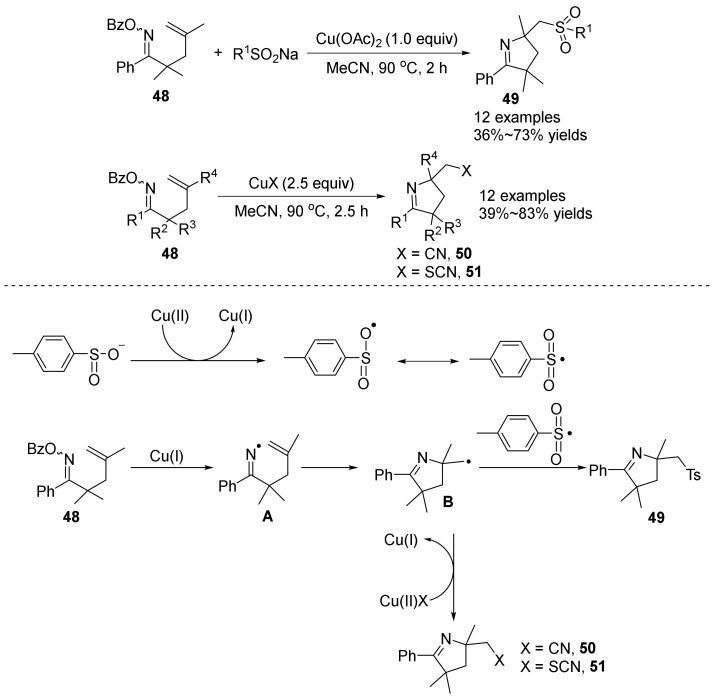
Cu-catalyzed N-O bond cleavage to 3,4-dihydro-2*H*-pyrroles and proposed mechanism.

In 2019, Zhu’s group [46] studied the iminyl radical-promoting iminosulfonylation **49**, iminocyanation **50** and iminothiocyanation **51** of γ,δ-unsaturated oxime esters **48** for the synthesis of multifunctional pyrroline (Figure 17). The reaction was carried out at 90 °C by using copper acetate as catalyst and acetonitrile as solvent, and a variety of multifunctional pyrroline compounds were constructed in good yield. A copper-initiated homolytic cleavage of the N-O bond to form an imine radical intermediate **A** was proposed. The introduction of the imine part in the molecule allows a series of bifunctional processes to be readily carried out: imine sulfonylation, imine cyanation and imine thiocyanation, thereby introducing useful functional groups (-SO_2_R, -CN, -SCN) into pyrroline. The introduction of multifunctional groups into pyrroline derivatives can further enhance the diversity of the molecule.

**Figure 18:**
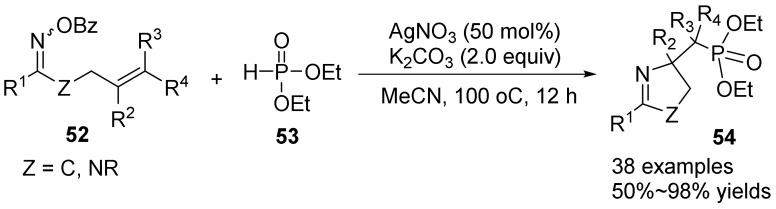
Ag-catalyzed N-O bond cleavage to phosphorylated pyrrolines.

In the same year, Zhu’s group [47] also reported the synthesis of phosphorylated pyrrolines **54** by silver-promoted cascade radical cyclization of γ, δ-unsaturated oxime esters **52** with phosphonate ester **53** (Figure 18). Using AgNO_3_ as a catalyst, K_2_CO_3_ as a base, acetonitrile as solvent, at 100 °C, various phosphorus-containing pyrrolines could be constructed directly at 100 °C in high yields without the need for oxidation conditions. The mechanism of the reaction is through a coupling between an iminyl radical intermediate and a phosphoacyl radical. This method provides a step-economic and redox-neutral method for obtaining a variety of phosphorylated pyrrolines. Through the deoxygenation process, a new large-volume trivalent phosphine ligand with pyrrole motif that is difficult to synthesize by other methods is obtained. The trivalent phosphine ligand can be used as chiral ligand and organic catalyst for asymmetric catalysis, and the obtained phosphorylated pyrrolines can also be used for further transformation.

**Figure 19:**
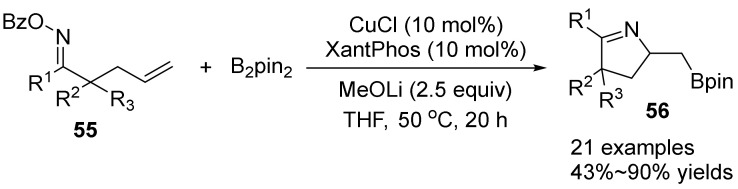
Cu-catalyzed N-O bond cleavage to form borated pyrrolines.

In 2020, Wu’s group [48] reported a copper-catalyzed aminoboration reaction of γ,δ-unsaturated aromatic oxime esters **55** with B_2_pin_2_ to synthesize a variety of borated pyrrolines **56** derivatives in high yields (Figure 19). Using B_2_pin_2_ as nucleophile, CuCl as catalyst, LiOMe as base, THF as solvent, a variety of borated pyrroline derivatives can be synthesized in high yield under mild conditions at 50 °C. This method is effective for the synthesis of useful boron-modified pyrrolines compounds and a new direction for the synthesis of functionalized indoles.

**Figure 20:**
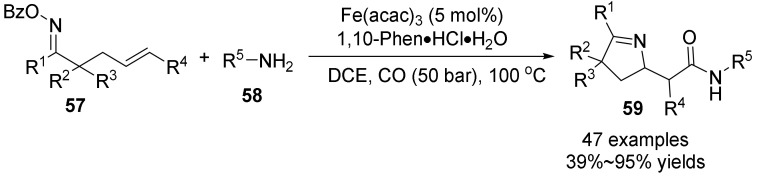
Fe-catalyzed N-O bond cleavage to form pyrrolines.

In 2020, Wu’s group [49] also studied the use of Fe(acac)_3_ as catalyst, 1,10-Phen·HCl·H_2_O as ligand, and DCE as solvent to achieve the efficient carbonyl cyclization of unactivated γ, δ-unsaturated aromatic oxime esters **57** and amines **58** to construct pyrrolines **59** with good functional group tolerance (Figure 20). The carbonylation reaction of α-unsubstituted γ, δ-unsaturated oxime esters with amines was achieved under alkaline conditions using inexpensive metal iron as catalyst, thus solving the problem that oxime esters with double-base no-/single-α substituents were difficult to react with amines due to the formation of C = C bonds by imine bonds. This strategy is an effective route to expand the scope of functionalized pyrrolines. A variety of oxime esters and amines have good compatibility with this scheme. The obtained carbonylation products, including cyclization and hydrogenation reactions, are successfully achieved in high yield.

**Figure 21:**
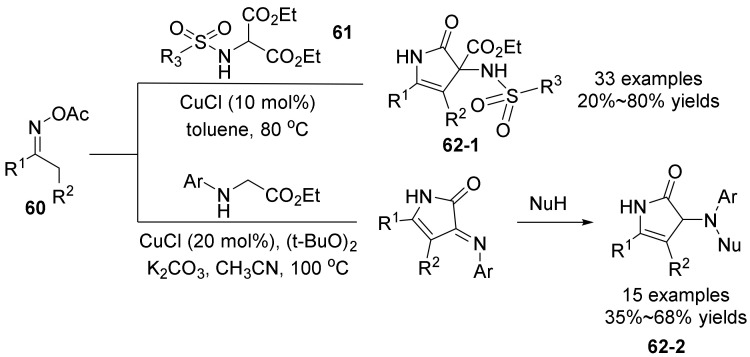
Cu-catalyzed N-O bond cleavage to 3-sulfamido **53-1**/imino-4-pyrrolone 4-pyrrolin-2-ones.

In 2020, Yang’s group [50] reported a copper-catalyzed cyclization of oxime acetates **60** with α-amino acid ester derivatives **61** to prepare 3-sulfamido **62-1**/imino-4-pyrrolone 4-pyrrolin-2-ones **62-2** bearing a 3-amino group (Figure 21). In this process, in the presence of a certain amount of CuCl, an electrophilic center was introduced on the α-carbon of the secondary amine, and by using oxime ester as an internal oxidant, the secondary amine was oxidized to an imine intermediate with two adjacent electrophilic centers. At the same time, the active 1,3-dinucleophilic species and 1,2-dielectrophilic species were generated, and the subsequent intermolecular nucleophilic cyclization reaction realized the efficient construction of 4-pyrroline-2-one derivatives. The functional group compatibility of the reaction is good, the operation is simple, the conditions are mild, and the starting material is easy to obtain, making this method very attractive.

**Figure 22:**
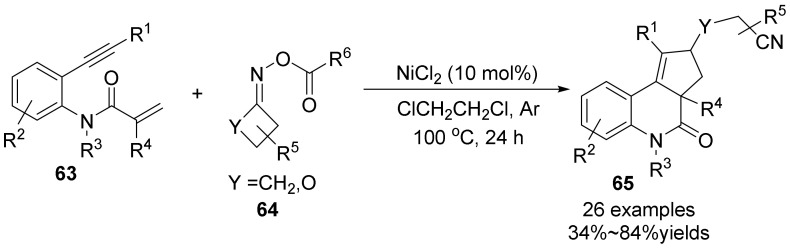
NiCl_2_-promoted [2+2+1] carboannulation of 1,7-enynes with cycloketone oximes.

Our group disclosed a NiCl_2_-promoted [2+2+1] carboannulation of 1,7-enynes **63** with internally oxidative cyclobutanone oximes to form 4*H*-cyclopenta[*c*]quinolin-4-ones **65** with cyano group by using cyclobutanone oxime esters **64** as a *n*-butyl radical precursor and internal oxidants (Figure 22) [51]. Under the optimal conditions, a variety of functionalized cyclobutanone oxime esters were smoothly converted into corresponding alkyl radical by the N-O bonds and C-C bonds cleavage. The reaction is achieved by continuous radical addition/cyclization/1,5-hydrogen migration and recyclization to afford the desired products in satisfactorily yields.

**Figure 23:**
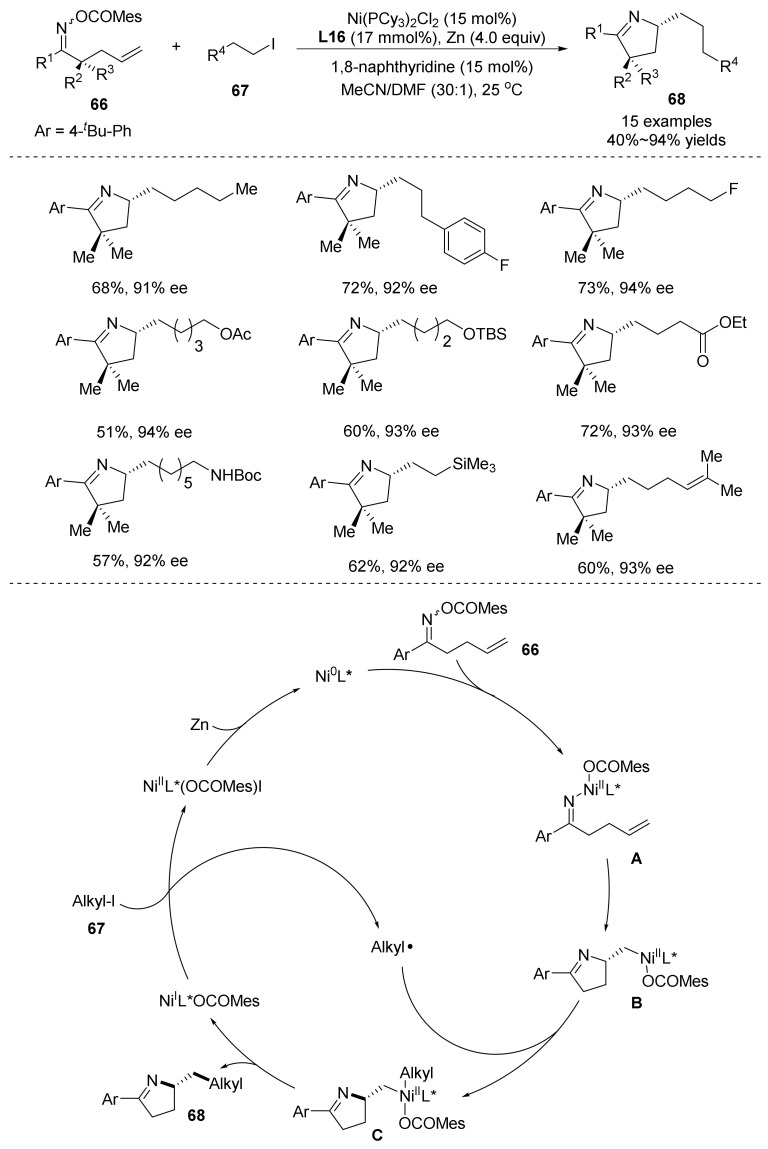
Ni-catalyzed N-O bond cleavage to form chiral pyrrolines.

In 2022, Shu’s group [52] reported the nickel-catalyzed enantioselective iminoalkylation of olefinic oxime esters **66** with alkyl iodides **67** (Figure 23). This method uses a newly defined pyridine bis-(oxazoline) ligand to avoid the *β*-H elimination of the cyclization intermediate, resulting in a Heck product that can highly enantioselectively generate chiral pyrrolines **68**. This work demonstrates the possibility of difunctionalization of the enantioselective cross-electrophiles of olefins by heterocyclization, thus providing an opportunity to obtain new chiral scaffolds that represent the first highly enantioselective aza-Heck/cross-coupling sequence, and therefore also establish a new platform for asymmetric aza-Heck and cross-coupling sequences. The functional group compatibility of the reaction is good, the coupling of secondary and primary alkyl iodides is good, and a few tertiary alkyl iodides can also be carried out, which improves the diversity of molecular structure.

The authors studied the reaction mechanism in depth and proposed the possible reaction mechanism for the formation of poly-substituted pyrroline compounds (Figure 23): First, the Ni(I)L*(OCOMes) react with alkyl iodide to produce the alkyl radical and Ni(II) intermediates. Ni(II)L*(OCOMes)I was reduce to Ni(0) by Zn dust. Then, complex **A** was prepared by the oxidative addition of oxime ester with Ni(0) catalyst. The intermediate **B** was obtained by selective migration of intermediate **A**. Then, alkyl radical, was produced by alkyl iodide added to the intermediate **B** to afford the intermediates **C**, and then the target product was obtained by reduction elimination.

**Figure 24:**
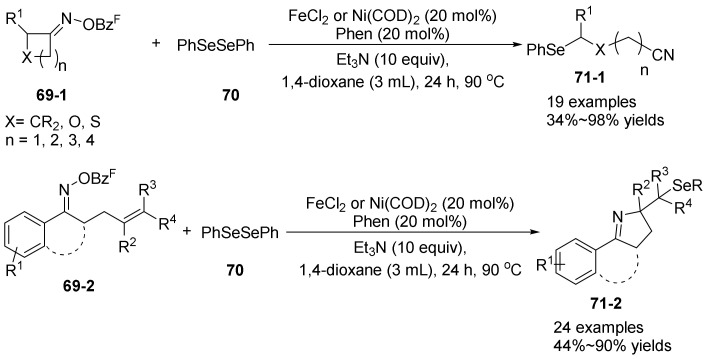
Ni-catalyzed or Fe-catalyzed N-O bond cleavage to alkyl nitriles and pyrroline.

In 2022, Zhou’s group [53] carried out a nickel-and iron-catalyzed cascade free radical reaction of oxime esters with **69**. The author used aryl oxime esters **69-2** to react with 1,2-diphenyldiselane **70** to form pyrroline compounds **71-2** (Figure 24), while for cycloketone oxime esters to react with 1,2-diphenyldiselane **70** to form alkyl nitriles **71-1**. A variety of functionalized pyrroline and alkyl nitriles were synthesized by this method. In this strategy, Ni(0)/Fe(II) catalysts can be used for the selenization of olefin-containing aryl oxime esters. Through Ni(0)/Fe(II) catalysis, N-O bond cleavage can generate nitrogen-centered radicals, and only a few examples of selenization processes through imine radical intermediates have been reported. Different substituents of oxime esters, Ni(0)/Fe(II) have different catalytic effects on them. For example, oxime esters containing heterocyclic substituents have better catalytic effect with Fe(II); the ortho-substituted aryl diselenide has a better catalytic effect with Ni(0). This method has high functional group tolerance and obtains a wide range of selenopyrrolines from readily available raw materials. These reactions may contribute to the development of free radical chemistry in organic synthesis and the application of organic selenides in biological applications.

### 2.4. The N-O Bond Cleavage of Oxime Esters to form Spirocyclic Compounds

**Figure 25:**
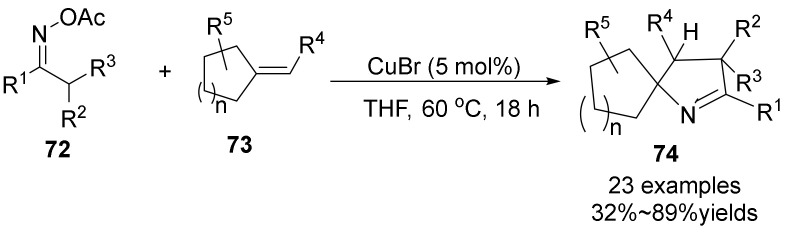
Cu-catalyzed N-O bond cleavage to form spirocyclic compounds.

In 2017, a Cu-catalyzed for the rapid formation of a broad structurally interesting spiropyrroline skeletons **74** was disclosed by Wei group [54] (Figure 25). Using the activated alkenes **73** and highly active oxime esters **72** can easily realize the cyclization reaction to form the spirocyclic compounds with broad substrate scope and good functional group tolerance in the presences of copper catalyst. Notably, this method can also be expanded well, such as the late modification of bioactive pregnenolone derivatives. The mechanistic investigation suggests that the reactions proceed through a radical process.

**Figure 26:**
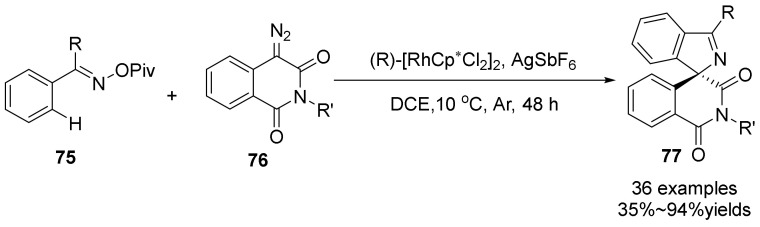
Rh-catalyzed N-O bond cleavage to form spirocyclic compounds.

In 2021, Li’s group [55] has exhibited a chiral Rh^III^-catalyzed asymmetric [4+1] spiroannulation of O-pivaloyl oximes **75** with α-diazo homophthalimides **76** with the aid of AgSbF_6_ (Figure 26). The method can achieve the asymmetric synthesis without oxidants and bases through N-O bond cleavage of oximes by using the α-diazo homophthalimides. The chiral rhodium catalyst can achieve both C-H activation and chiral control in this reaction. leading to the formation of C(sp^3^)-C(sp^2^) and C(sp^3^)-N bonds. The authors also obtained k_H_/k_D_ = 2.2 under standard conditions using a kinetic isotope effect, suggesting that the ortho C-H activation event may be involved in the turnover-limiting step. The reaction proceeded with high efficiency and features broad substrate scope, mild reaction conditions, and high-to-excellent enantioselectivities.

### 2.5. The N-O Bond Cleavage of Oxime Esters to form Pyridine Derivatives

**Figure 27:**
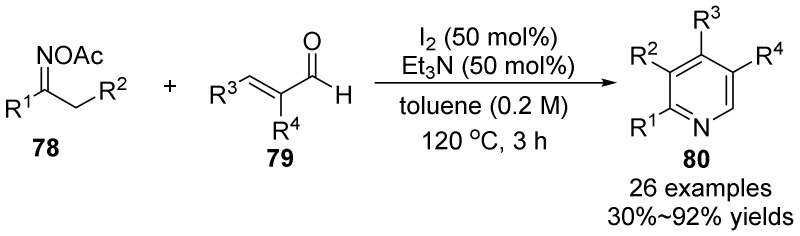
Metal-free-catalyzed N-O bond cleavage to polysubstituted pyridines.

In 2016, Deng’s group [56] developed a transition metal-free catalytic oxime esters **78** and acrolein **79** reaction to synthesize polysubstituted pyridines **80** (Figure 27). This method is different from the traditional transition metal catalytic method, but through the combination of iodine and triethylamine, the oxime ester is effectively reduced, the N-O bond of the oxime ester is excited, and the imine radical is generated and the acrolein is efficiently reacted to form functionalized pyridine. This method can reduce the damage to the environment, has high chemical selectivity and good tolerance to a wide range of functional groups, and represents a new way to synthesize oxime N-heterocycles based on metal-free systems.

**Figure 28:**
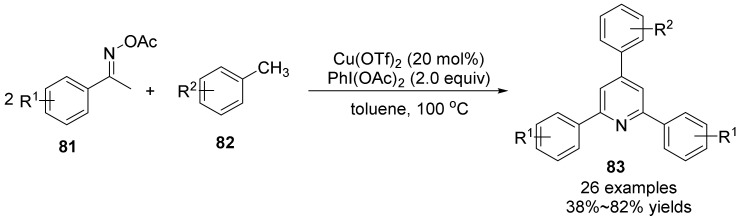
Cu(II)-catalyzed N-O bond cleavage to polysubstituted pyridines.

In 2016, Chen’s group [57] realized an efficient and concise method for the synthesis of polysubstituted pyridines **83** by copper-catalyzed oxidative coupling of C(sp_3_)-H acetate oxime esters **81** with toluene derivatives **82** (Figure 28). The oxidative cleavage of the C(sp^3^)-H bond was catalyzed by Cu(OTf)_2_ and oxidized by PhI(OAc)_2_. In addition, the author also proposed two alternative transformation methods to introduce the reaction of benzylamine and toluenesulfonyl hydrazone with oxime acetate, which enriched the diversity of substituted pyridine synthesis methods. These preparation methods of polysubstituted pyridines have the advantages of easy availability of raw materials, simple operation, cheap catalysts and mild conditions, and provide a highly flexible and simple preparation method of substituted pyridines.

**Figure 29:**
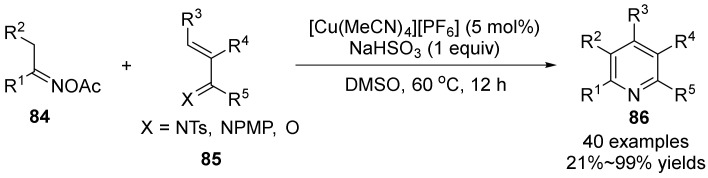
Cu-catalyzed N-O bond cleavage to polysubstituted pyridines.

In 2017, Yoshikai’s group [58] reported a copper-catalyzed condensation reaction of oxime acetate **84** with *α,β*-unsaturated imines **85** for regioselective synthesis of highly substituted pyridines **86** (Figure 29). The reaction conditions of this method are simple and mild. Under the catalysis of copper salt, the unactivated *α,β*-unsaturated imines can be reacted, and it has high regioselectivity and high functional group compatibility for asymmetric oxime acetate, thus resulting regioselectively synthesize a wide range of polysubstituted pyridines, including pyridines that are not easily obtained by conventional condensation methods.

**Figure 30:**
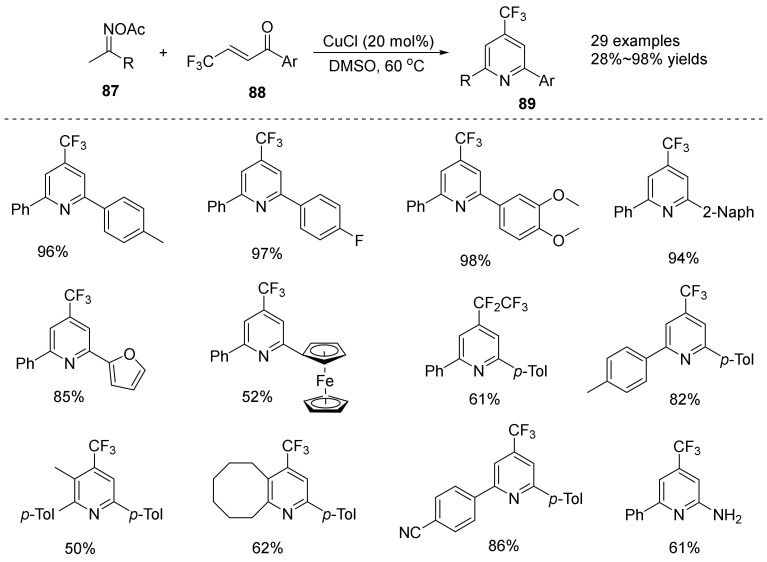
Cu-catalyzed N-O bond cleavage to trifluoromethylate pyridine.

In 2018, Li’s group [59] reported a copper-catalyzed redox divergent [3+3] coupling reaction of oxime esters **89** with β-trifluoromethyl ketones **88** and acrylates **87**. Under different conditions, trifluoromethylated/difluoromethylated pyridine (Figure 30) can be provided with *β*-trifluoromethyl ketones and acrylates, respectively. The reaction has a wide range of substrates, regional/redox selectivity, mild conditions, and has potential application prospects in the synthesis of fluorine-containing nitrogen heterocyclic drugs.

**Figure 31:**
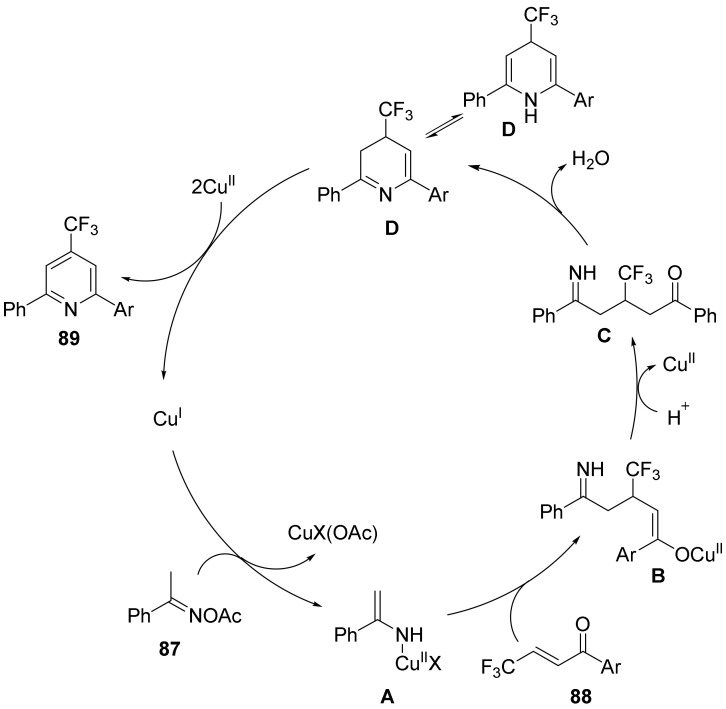
Proposed mechanism for the synthesis of fluoropyridine.

The reaction mechanism was investigated in depth and a possible reaction mechanism for the formation of fluoropyridine compounds was proposed (Figure 31): Cu(I) was oxidized to **A** by oxime ester, followed by the reaction of **A** with **79** to form intermediate **B.** Hydrolysis of **B** leads to **C**, followed by dehydration and cyclization to form intermediate **D,** with product **89** formed by **D** with aid of Cu (II).

**Figure 32:**
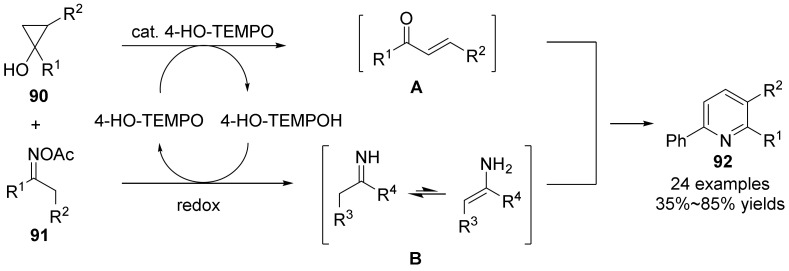
4-HO-tempo -catalyzed N-O bond cleavage to form pyridines.

In 2019, Han’s group [60] carried out a redox cyclization reaction of cyclopropanol **90** and oxime acetate **91** catalyzed by 4-HO-tempo to produce pyridine derivatives **92** in good yield (Figure 32). Under the catalysis of TEMPO, the key intermediates of α, β-unsaturated ketones **A** and imines **B** were obtained by cyclopropanol and oxime acetate, respectively. Then, [3+3] cyclization was carried out to obtain dihydropyridine, which was further oxidized to pyridine products. This method has the advantages of good functional group tolerance, high chemical selectivity, wide substrate range and good compatibility with natural products and drug molecular frameworks. This method is the first example of TEMPO-catalyzed redox reaction, which broadens the field of TEMPO catalysis, and establishes a new and efficient synthesis of pyridine derivatives under metal-free conditions.

**Figure 33:**
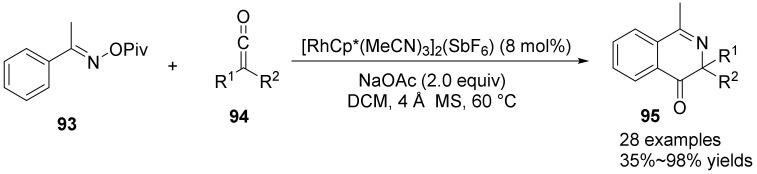
Rh(III)-catalyzed N-O bond cleavage to isoquinolinones.

In 2018, Li’s group [61] studied the [4+2] cyclization reaction of O-pivaloyl oxime esters **93** with ketenes **94** under Rh (III)-catalyzed redox neutral conditions to synthesize isoquinolinones **95** containing quaternary carbon stereocenters (QCSC) (Figure 33). In this strategy, N-OPiv, as an oxidizing group with some coordination, saturates the coordination sphere, acts as a key factor in inhibiting the proton decomposition of Rh-C (alkyl) bonds, and accelerates the reduction elimination to form a quaternary carbon-containing stereocenter (QCSC), which can be applied to the synthesis of complex bioactive products.

## 3. The N-O Bond Cleavage of Hydroxylamine to Construct N-Heterocycles

**Figure 34:**
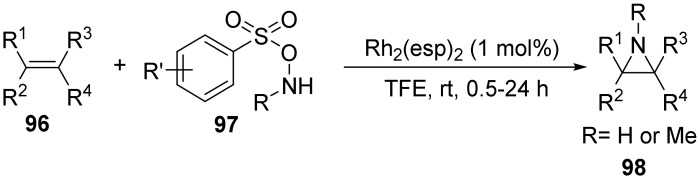
Rh(II)-catalyzed N-O bond cleavage to unactivated aziridines.

In 2018, Jat’s group [62] reported the Rh(II)-catalyzed amination of olefins **96** with O- (sulfonyl) hydroxylamine **97** to synthesize unactivated aziridines **98** in good or excellent yields (Figure 34). The reaction does not require alkali as an additive, nor does it require column chromatography. After this reaction, it is possible to obtain the target product in high purity after aqueous treatment, avoiding the possibility of aziridine ring opening during silica gel purification. There is good regioselectivity for this reaction, no interfering by-products are produced, and it is also well tolerated by highly reactive and unstable functional groups.

**Figure 35:**
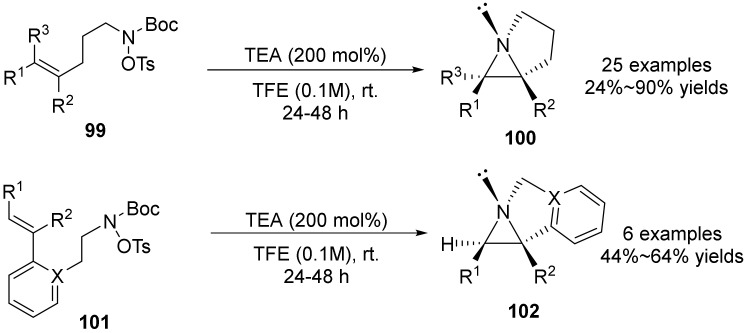
Metal-free-catalyzed N-O bond cleavage to unactivated aziridines.

In 2018, Bower’s group [63] reported that under metal-free reaction conditions, the in-situ deprotection of N-Boc hydroxylamine **99** activated by O-Ts of difunctional amino reagents can trigger the intramolecular N-heterocyclization of N-chain olefins, and N-alkylation proceeds via the N-heterocyclization of olefins **101** in the presence of TEA (triethanolamine) (Figure 35). The olefin amino reagent carried out an intramolecular stereoselective olefin N-heterocyclization reaction to obtain N-alkylated products in good yield. After removing the protective group of the amino reagent, the author found that the transition state is very similar to the helical’ butterfly’ transition state of m-CPBA-intermediate alkene epoxides, confirming that this is a diastereospecific Aza-Prilezhaev type reaction.

**Figure 36:**
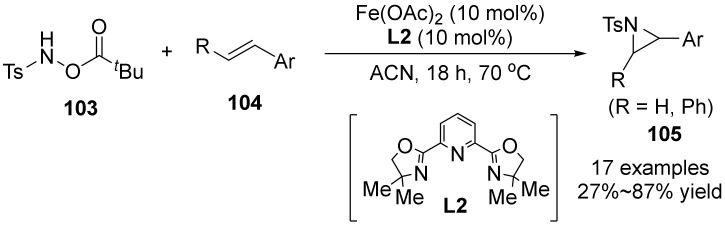
Fe-catalyzed N-O bond cleavage to aziridines.

In 2021, Berhal’s group [64] reported the first intermolecular N-heterocyclization of hydroxylamine derivatives **103** with olefins **104**, affording various aziridines **105** in the simple ligands and Fe(OAc)_2_ (Figure 36).The reaction does not require dangerous reagents and reaction conditions, and does not affect the efficiency of the N-heterocyclization reaction at a catalyst dosage of 5 mol%. It is effective for both styrene and aliphatic olefins, and the released carboxylic acid can be recovered, which enhances the sustainability and atomic economy of the method.

**Figure 37:**
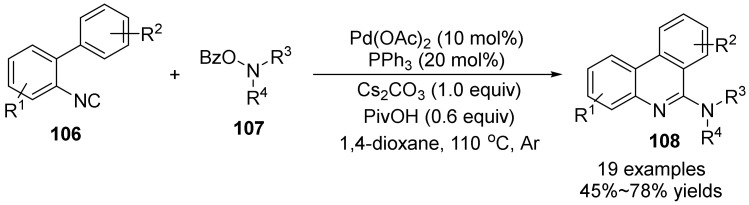
Pd-catalyzed N-O bond cleavage to amino-substituted phenanthridines.

In 2017, Zhu’s group [65] carried out palladium-catalyzed synthesis of amino-substituted N-heterocycles **108** from phthaloyl hydroxylamines **107** and isocyanoaromatics **106** (Figure 37). The use of O-benzoyl hydroxylamine as an oxidizing amino source to generate amino Pd(II) intermediates, isocyanate migration insertion and intramolecular C(sp^2^)-H activation, the reaction introduces amino groups in the formation of C-N bonds and C-C bonds and the formation of heterocycles. This strategy provides an efficient and practical method for the construction of amino-substituted six-membered N-heterocyclic compounds.

## 4. Conclusions and Outlook

With the development in the field of transition metal catalysis and radical chemistry, the synthesis of N-heterocycles by using oxime and hydroxylamines has become one of the most powerful tools in organic synthesis. In the past few years, the strategy of nitrogen-oxygen bond cleavage of oxime and hydroxylamines has been widely used for the synthesis of various structurally diverse *N*-heterocycles. Using oximes and hydroxylamines through N-O bond cleavage have also been well-established for efficient access to the *N*-heterocycles, such as aziridines, *β*-lactam, 1*H*-indazoles, oxazoles, pyrrolines, pyrroles, pyridines, and other cyclic compounds. In the past few years, the synthesis of *N*-heterocycle by using oximes and hydroxylamines as nitrogen sources are summarized and discussed according to different nitrogen sources and heterocyclic types. Recently, the construction of heterocyccles by using the oxidation characteristics of oxime esters is more efficient and sustainable. Most of these reactions are usually conducted under cheap transition metal catalyst and oxygen, allowing the construction of N-heterocycles with good functional group tolerance and more diverse transformation. It is worth noting that the radical strategy shows more powerful prospects than the conventional transition metal catalytic strategy [9,66], allowing the development dual catalysis systems, such as synergistic copper and photoredox catalysis and synergistic nickel and photoredox catalysis [67,68,69,70]. In particular, the iminyl radicals as unstable nitrogen radicals can be transformed more often to synthesize complex and diverse N-heterocycles [71,72,73]. Despite these advances, there are still many directions for improvement and further development in this field. For example, the enantioselective synthesis of N-heterocycles is still limited to transition metal catalysis, and radical strategies are less well studied. We hope that this review provides a new perspective on this field, and also provides a reference to develop environmentally friendly and sustainable methods.

## Data Availability

Not applicable.
